# Supplementary material for: The Construction and Exploration of a Comprehensive MicroRNA Centered Regulatory Network in Foxtail Millet (Setaria italica L.)
Source: Front Plant Sci. 2022 May 6;13:848474. doi: 10.3389/fpls.2022.848474 (PMC9121102; doi:10.3389/fpls.2022.848474)
Supplement: Supplementary file 7 [file Data_Sheet_7.zip › Sit-miR398a_Seita.4G226500.1_1157_TPlot.pdf]

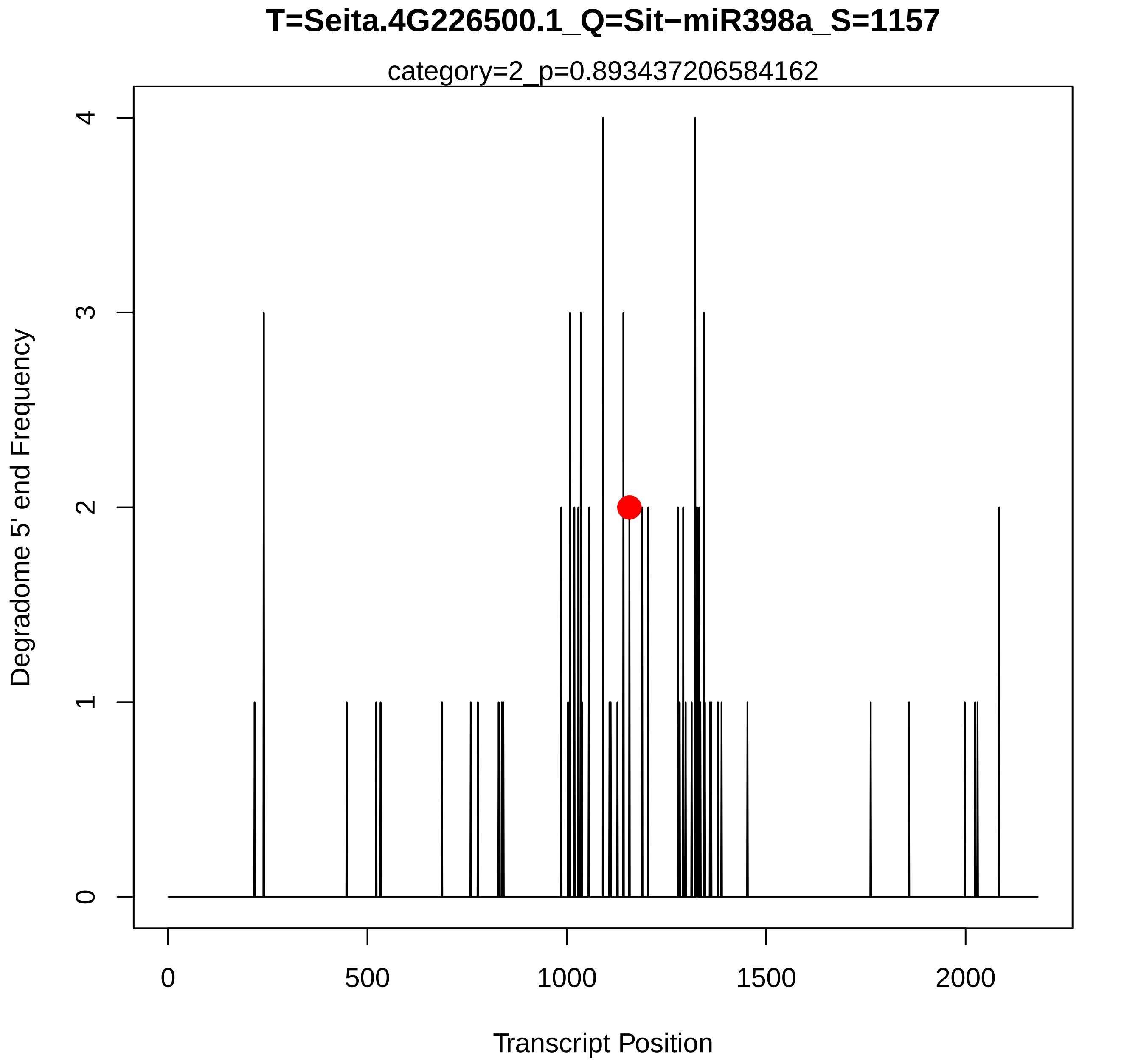
T=Seita.4G226500.1_Q=Sit−miR398a_S=1157
category=2_p=0.893437206584162
Degradome 5' end Frequency
Transcript Position
0
1
2
3
4
0
500
1000
1500
2000
